# Images in Pediatric Neurosurgery: Occult Intraosseous Dermoid Cyst at the Nasofrontal Junction

**DOI:** 10.1159/000528440

**Published:** 2022-11-30

**Authors:** Yusuke S. Hori, John S. Albanese, John G. Meara, Mark R. Proctor

**Affiliations:** Department of Neurosurgery and Plastic and Oral Surgery, Boston Children's Hospital, Harvard Medical School, Boston, Massachusetts, USA

An 8-year-old previously healthy female was initially found to have a small pit on her nasal dorsum. The lesion developed local infection and she was initially treated with antibiotics 2 years prior to the current presentation. The lesion was diagnosed as a dermal sinus tract, and surgical removal was conducted at an outside hospital. In retrospect, the preoperative work-up imaging showed an occult intraosseous nasal bone extension; however, this was not appreciated at the initial surgery. She experienced repeat infections and underwent a second surgery with exploration under the nasal bones; however, the patient experienced recurrent post-operative local infections. The family presented to our institution for a second opinion.

On review, the head CT demonstrated an intraosseous cystic lesion completely hidden in the nasal bone (Fig. 1a). The lesion was consistent with dermoid cyst on MRI (Fig. 1b, c). The patient proceeded with a third surgery for complete removal of the lesion via an extended vertical nasal incision. The nasal bones were removed in their entirety, the occult dermoid cyst with a small tract was located in the nasal bones, and the undersurface of the bones was completely debrided. No intracranial extension was observed after careful investigation of the skull base. Particulate corticocancellous bone was used with fibrin sealant to reconstruct the defect. The nasal bones were then replaced (Fig. 1d). The pathology results were consistent with a dermoid cyst. The post-operative course was uncomplicated and she has not had a recurrence after the third surgery.

To date, no previous reports have documented a case with intraosseous dermoid cyst which was completely hidden in the nasal bone. An extension of the dermoid cyst below the nasal bone has been identified in 10% of patients in a large series of nasal dermoid cysts [1], but these are generally easily identifiable and connected to the tract. Ni et al. [2] previously reported several dermal cyst cases with an extension through the nasal bone, while these cases had primary lesions in the subcutaneous region and an easily identifiable connection to the cyst. Our images interestingly illustrate the nasal dermoid cyst extending into the nasal bone at the nasofrontal junction without detectable extraosseous subnasal bone extension on the imaging. Several studies have reported surgical techniques to facilitate the complete resection of the nasal dermoid cyst [3, 4]. This case, despite an exploration under the nasal bones, initially received incomplete resection and experienced multiple infections because of failure to appreciate the portion hidden in the nasal bones. Our case was successfully treated with ostectomy of nasal bones without recurrence and complications. This procedure allows unobstructed visualization of the entire cyst leading to the complete removal of the lesion. This is an instructive case to show that portions of the cyst may remain hidden and lead to recurrent infection, and complete resection with sufficient exposure of the entire lesion is needed to successfully treat this rare condition.

## Statement of Ethics

Written informed consent was obtained from the parent of the patient for publication of the details of their medical case and any accompanying images. This study protocol was reviewed and the need for approval was waived by the Boston Children's Hospital Institutional Review Board.

## Conflict of Interest Statement

The authors have no conflicts of interest to declare.

## Funding Sources

The authors received no financial support for the research, authorship, and publication of this article.

## Author Contributions

Design of the manuscript: Yusuke S. Hori, John G Meara, and Mark R. Proctor; acquisition, analysis, and interpretation of data: Yusuke S. Hori, John S. Albanese, John G. Meara, and Mark R. Proctor; drafting the manuscript: Yusuke S. Hori; and revising the manuscript: Yusuke S. Hori, John G. Meara, and Mark R. Proctor.

## Data Availability Statement

All data generated or analyzed during this study are included in this article. Further inquiries can be directed to the corresponding author.

## Figures and Tables

**Fig. 1:**
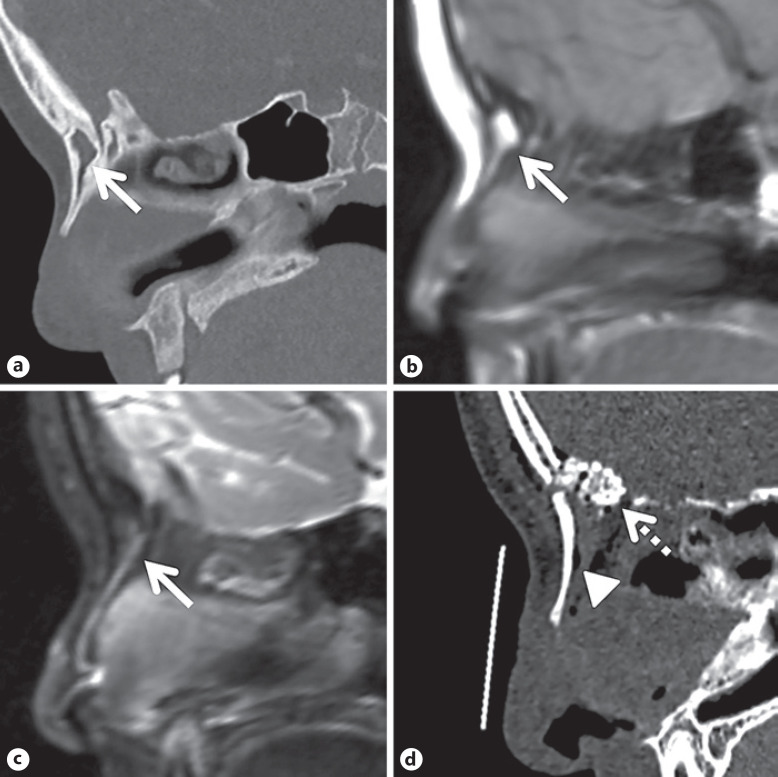
**a** Sagittal head CT showing intraosseous cystic lesion completely hidden in the nasal bone. **b** T1-weighted brain magnetic resonance imaging showed hyperintense cystic contents. **c** Short tau inversion recovery sequence demonstrated fat-suppressed signals in the corresponding area. **d** Post-operative head CT demonstrating replaced nasal bone (arrowhead) and particulate corticocancellous bone (dashed arrow) reconstructing the skull base defect.
